# MiR-144-3p inhibits the proliferation and metastasis of lung cancer A549 cells via targeting HGF

**DOI:** 10.1186/s13019-022-01861-3

**Published:** 2022-05-14

**Authors:** Guiju Fang, Canhui Zhang, Zhixin Liu, Zhiwen Peng, Meiyan Tang, Qing Xue

**Affiliations:** Department of Respiratory Medicine, Ningde Municipal Hospital of Ningde Normal University, Ningde, 352100 People’s Republic of China

**Keywords:** Molecular mechanism, Lung cancer, microRNA-144-3p, HGF

## Abstract

**Aim:**

MicroRNAs have been confirmed as vital regulators in gene expression, which could affect multiple cancer cell biological behaviors. This study aims to elucidate the molecular mechanism of miR-144-3p in lung cancer cellular proliferation and metastasis.

**Methods:**

MiR-144-3p expression in lung cancer tissues and cell lines was detected by qRT-PCR. HGF was predicted as the target gene of miR-144-3p using TargetScan and dual luciferase reporter assay. Immunohistochemistry and qRT-PCR were used to explore the impacts of HCF on lung cancer tissues and cell lines. Impacts of miR-144-3p and HGF on cancer cellular proliferation, migration and invasion were elucidated by CCK-8, Flow cytometry, Transwell invasion and Wound-healing assay. Moreover, nude mouse xenograft model was established to evaluate the effects of miR-144-3p on lung cancer cells.

**Results:**

MiR-144-3p exhibited a reduction in both lung cancer tissues and cell lines. HGF was a direct target of miR-144-3p. In contrast to the miR-144-3p expression level, HGF showed a higher level in lung cancer tissues and cell lines. Overexpression miR-144-3p suppressed A549 and NCI-H1299 cell proliferation and metastasis, whereas this was reversed by HGF. MiR-144-3p exhibited an inhibitory effect on A549 cell-induced tumor growth of nude mice.

**Conclusions:**

This study reveals miR-144-3p/HGF axis may be involved in the suppression of lung cancer cellular proliferation and development, and miR-144-3p may function as a potential therapeutic target in lung cancer treatment in the future.

## Introduction

MicroRNAs (miRNAs/miRs) are short noncoding RNAs, which can post-transcriptionally regulate gene expression via binding with 3′-untranslated region of mRNAs [1, 2]. Mounting evidence indicates that miRNAs function as endogenous agents of multiple target genes in regulating molecular and cellular processes, which includes cell cycle, proliferation, growth and apoptosis [3–7]. Thus, it is not surprising to find miRNAs also widely involved in carcinomas development. As previously reported, dysregulation of miRNAs is closely correlated with cancer initiation and progression [8, 9]. However, the precise role of miR-144-3p in lung cancer cells still needs to be elucidated.

Lung cancer is responsible for a leading cause of death across the world [10]. Although advances in therapeutic strategies and genomics are encouraging, the lung cancer-related mortality is still rising. Given that the precise mechanisms of various types of lung cancer have not been totally characterized yet, there is an urgent need to explore the molecular pathogenesis and identify an effective bio-marker for lung cancer therapy. Dysregulated miRNAs are associated with lung cancer [11]. MiR-145 has been demonstrated to be a potential agent in suppressing lung cancer growth through targeting the Wnt/ß-catenin signal transduction cascade [12]. MiR-15a/16 has also been reported to behave as a potential novel therapeutic target for attenuating radio-resistance during lung cancer treatment [13]. However, the detailed role of miR-144-3p and its molecular mechanisms in lung cancer are not fully understood and revealed.

MiR-144-3p, situated at NC_000017.11, was a newly identified microRNA and reported to undertake pivotal roles in the progression of human cancers [14]. Recently, accumulating research has emphasized a vital and potential role for miR-144-3p to play in tumorigenesis. MiR-144-3p exhibited poor expression in various types of malignant tumor, including osteosarcoma and glioblastoma [15, 16]. In addition, miR-144-3p had a correlation with gastric cancer [17]. Furthermore, miR-144-3p showed an inhibitory effect on liver cancer cellular proliferation and metastasis via targeting E2F3 [18]. A previous study showed that miR-144-3p could suppress NSCLC progression via inhibiting CEP55 expression, but much more complicated mechanism of miR-144-3p is still unknown [19]. In our study, we investigated the potential role of miR-144-3p in lung cancer. We also assessed the implications of dysregulated miR-144-3p expression in lung cancer cellular processes, and explored the molecular mechanism involved.

## Materials and methods

### Clinical tissue specimen

58 patients (42 males and 16 females; aged 58.69 ± 12.71) with lung cancer were enrolled in this study. The inclusion criteria: (1) The cancer is confirmed by postoperative pathological examination; (2) all patients have not undergone preoperative radiotherapy or chemotherapy. The exclusion criteria: (1) Patients with other types of tumors; (2) patients have undergone preoperative radiotherapy or chemotherapy. After lung tumor tissues and adjacent normal tissues were obtained and underwent histological diagnosis, tissues were frozen in liquid nitrogen for subsequent assays. This study had received the approval from the Ethics Committee of Ningde Municipal Hospital of Ningde Normal University. Written informed consent for tissue usage was signed by patients.

### RT-qPCR

Extracting total RNA from clinical specimens and from various lung cancer cell lines was performed as previously described [11]. MiRNA expression was determined using the miRNA-specific TaqMan MiRNA Assay Kit (Applied Biosystems; Thermo Fisher Scientific, Inc., Waltham, MA, USA) following the protocol of the manufacturer. Detailed procedure was in accordance with a previous study [20]. All samples were evaluated in triplicate. The primer sequences used were shown as following:

miR-144-3p-F: 5′-TCCGATCATGTAGTAGATATTGACAT-3′,

miR-144-3p-R: 5′-GTGCAGGGTCCGAGGT-3′,

HGF-F: 5′-GTGTGCCACAACTCACAACTA-3′,

HGF-R: 5′-GGTCCTGGGTATTGGAGCA-3′,

U6-F: 5′-TCCGATCGTGAAGCGTTC-3′,

U6-R: 5′-GTGCAGGGTCCGAGGT-3′,

GAPDH-F: 5′-TGGACATCCGCAAAGACC-3′,

GAPDH-R: 5′-GGGACTTCCTGTAACGC-3′.

### Cell transfection

Purchasing lung cancer cell lines from the American Type Culture Collection (ATCC, Manassas, VA, USA), the cells were maintained in Dulbecco's Modified Eagle Medium (DMEM) that contained fetal bovine serum (10% FBS) and cultured at 37 °C under the conditions of 5% CO_2_ and saturated humidity. An inverted microscope was adopted to observe the growth of lung cancer cells. As A549 cells andNCI-H1299 cells reached 70–80% confluence, they were harvested with trypsin and sub-passaged. Lipofectamine 2000 (Invitrogen; Thermo Fisher Scientific, Inc.) was adopted to proceed transfection following the protocol of manufacturer. A549 and NCI-H1299 cell lines were divided into four groups: control group, miR-144-3p group, miR-144-3p + vector group and miR-144-3p + HGF group. A549 and NCI-H1299 cells were transfected with scrambled control mimics, miR-144-3p mimic, respectively or co-transfected with miR-144-3p plus vector or miR-144-3p plus HGF. Used sequences were shown as below:

miR-NC: 5′-UCACAGUGAACCGGUCUCUUU-3′,

miR-144-3p mimics: 5′-GGAUAUCAUCAUAUACUGUAAG-3′.

### Luciferase reporter assays

Online databases, which included EIMMO and miRanda-mirSVR (microRNA.org), were used to predict the potential target genes of miR-144-3p. Reporter vectors that contained wild-type (WT) or mutant (Mut) HGF3′-untranslated region (UTR) were constructed. A549 cells were co-transfected with miR-144-3p mimics or negative control miRNA mimics (pMIR-Control), together with reporter vectors. According to the recommendations of the manufacturer, Luciferase activity was determined through the Dual-Luciferase Reporter Assay System (Promega, Madison, WI, USA).

### Western blot

All of protein coming from transfected cells (about 2 × 10^6^ cell each group) was extracted and subjected to sodium dodecylsulfate polyacrylamide gel electrophoresis (SDS-PAGE), then transferred on polyvinylidene difluoride membranes. This assay was carried out as previously described [11] with primary antibodies against HGF (1:500, Sigma-Aldrich; Merck KGaA). The protein samples were then cultured together with a secondary antibody (Abcam, Cambridge, MA, USA). GAPDH was chosen as the internal control.

### Cell proliferation assay

The Cell Counting Kit-8 (CCK-8) assay was performed with an aim of detecting lung cancer cell proliferation. Briefly, transfected A549 cells (100 μl/well) and NCI-H1299cells (100 μl/well) were inoculated into 96-well plates with culturing condition maintained at 37 °C and 5% CO_2_. The proliferation of A549 and NCI-H1299 cells was measured at 3 days adopting CCK-8, following the protocol of the manufacturer. The optical density (OD) value at 490 nm was measured using an automatic microplate reader. The assay was performed in triplicate.

### Transwell invasion

Detecting invasions of A549 and NCI-H1299 cells was achieved by Transwell invasion assay. Briefly, transfected A549 and NCI-H1299 cells were harvested and propagated (about 2 × 10^6^ cells each group), then inoculated to the top chamber (10^5^ cells/chamber) and maintained under standard conditions for 24 h. Subsequently, transmigrated-cells were fixed and dyed, and counted using an inverted microscope. The assay was performed in triplicate; five random visual fields were selected for each chamber.

### Wound-healing assay

Detecting migration of A549 cells and NCI-H1299 cells was achieved by Wound-healing assay. Briefly, A549 and NCI-H1299cells were seeded into 6-well plates after transfection (2 × 10^5^cells/well) and cultured under standard conditions. A wound was produced by a 200 μl sterile pipette tip to scrape monolayer-cell. Fresh medium was added and the plate was cultured for 24 h. Using an inverted microscope to gain the images, the area covered by cells due to migration into the artificial wound was observed.

### Cell cycle measurement

Flow cytometry was adopted to observe cell cycle distribution. Transfected cells (2 × 10^6^ cell each group) were suspended in PBS and stained with 10 μl Annexin V-FITC as well as 5 μl PI reagents about 15 min away from light at room temperature. Adopting FACSCalibur flow cytometer (BD Biosciences, Franklin Lakes, NJ, USA), we measured cell proportion in S-phase, followed by analyzing the obtained data with Cell Quest software (BD Biosciences).

### Mouse xenograft model in vivo

Establishing a mouse xenograft model to explore the functional role of miR-144-3p in vivo, A549 cells were allowed to be plated in six-well plate, then transfected with miR-144-3p (miR-144-3p group) or control mimics (miR-NC group). Nude mice accepted the subcutaneous injection of these treated cells on the left flank (BALB/c, from Experimental Animal Center, Chinese Academy of Sciences). The above procedures were conducted based on the national (D.L.n.26, March 4, 2014) and international laws and policies (Directive 2010/63/EU). This study had received the approval from the Animal Experimental Ethics Committee, Ningde Municipal Hospital of Ningde Normal University. Mice were randomized into two groups with each group having 8 mice, which included control group (treated with control mimics) and miR-144-3p group (treated with miR-144-3p). When measuring tumor volume, the formula was: V = (L × W^2^) × 0.5, with L and W respectively representing the length and width. Mouse tumors were dissected out using a sterile scalpel, separated from skin carefully, weighed at day 21 post-inoculation, and then preserved in 4% paraformaldehyde for further analysis. The procedures were in accordance with the Guide for the Care and Use of Laboratory Animals (The 8th edition, NIH).

### Immunohistochemistry

Immunohistochemical (IHC) staining of tumor tissues was conducted as previously described [21]. An antibody against HGF protein (1:100, Boster, Hubei, China) was incubated with the tissue samples, and the stained samples were examined under a light microscope.

### Statistical analysis

Experimental data are presented as the mean ± standard deviation. When making multiple group comparisons, one-way analysis of variance was performed, while Student’s t-test was adopted to make a two-group comparison. It was considered to have statistical significance if *P* < 0.05.

## Results

### MiR-144-3p expression levels in lung tissue samples and various cell lines

**Fig. 1:**
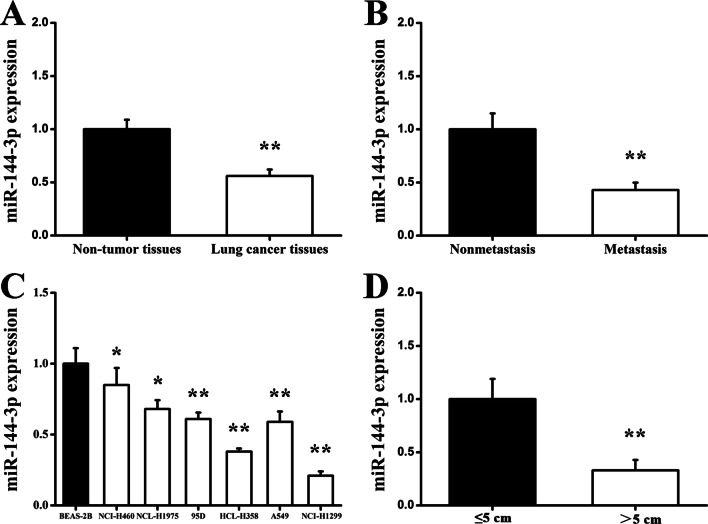
Expression levels of miR-144-3p in lung cancer tissues and various lung cancer cell lines. **a** RT-qPCR was used to analyze miR-144-3p expression in lung cancer tissues and matched normal tissues. **b** RT-qPCR was used to analyze miR-144-3p expression in metastatic tumor tissues and non-metastatic tumor tissues. **c** RT-qPCR was used to analyze the miR-144-3p expression in lung cancer cell lines and normal lung cell line. **d** RT-qPCR was used to analyze the miR-144-3p expression in tumor size of > 5 or ≤ 5 cm. *P < 0.05, **P < 0.01 versus control group

In order to explore the specific and potential role of miR-144-3p in lung cancer development, RT-qPCR was adopted to assess miR-144-3p expression in clinical specimens and various cell lines. Lung cancer tissues displayed attenuated miR-144-3p expression when compared to adjacent normal lung tissues (Fig. 1a). Additionally, miR-144-3p levels were markedly reduced in metastatic tumor tissues when making a comparison with non-metastatic tumor tissues (Fig. 1b). In lung cancer cell lines, RT-qPCR results indicated the miR-144-3p expression in six lung cancer cell lines was obviously and statistically decreased when compared with normal lung cells (Fig. 1c). Moreover, miR-144-3p levels in tumors > 5 cm in size were decreased in comparison to tumors ≤ 5 cm in size (Fig. 1d). Taken these results together, it is suggested that miR-144-3p could have a close link with lung cancer development and its progression.

### MiR-144-3p regulates HGF via directly targeting HGF

**Fig. 2:**
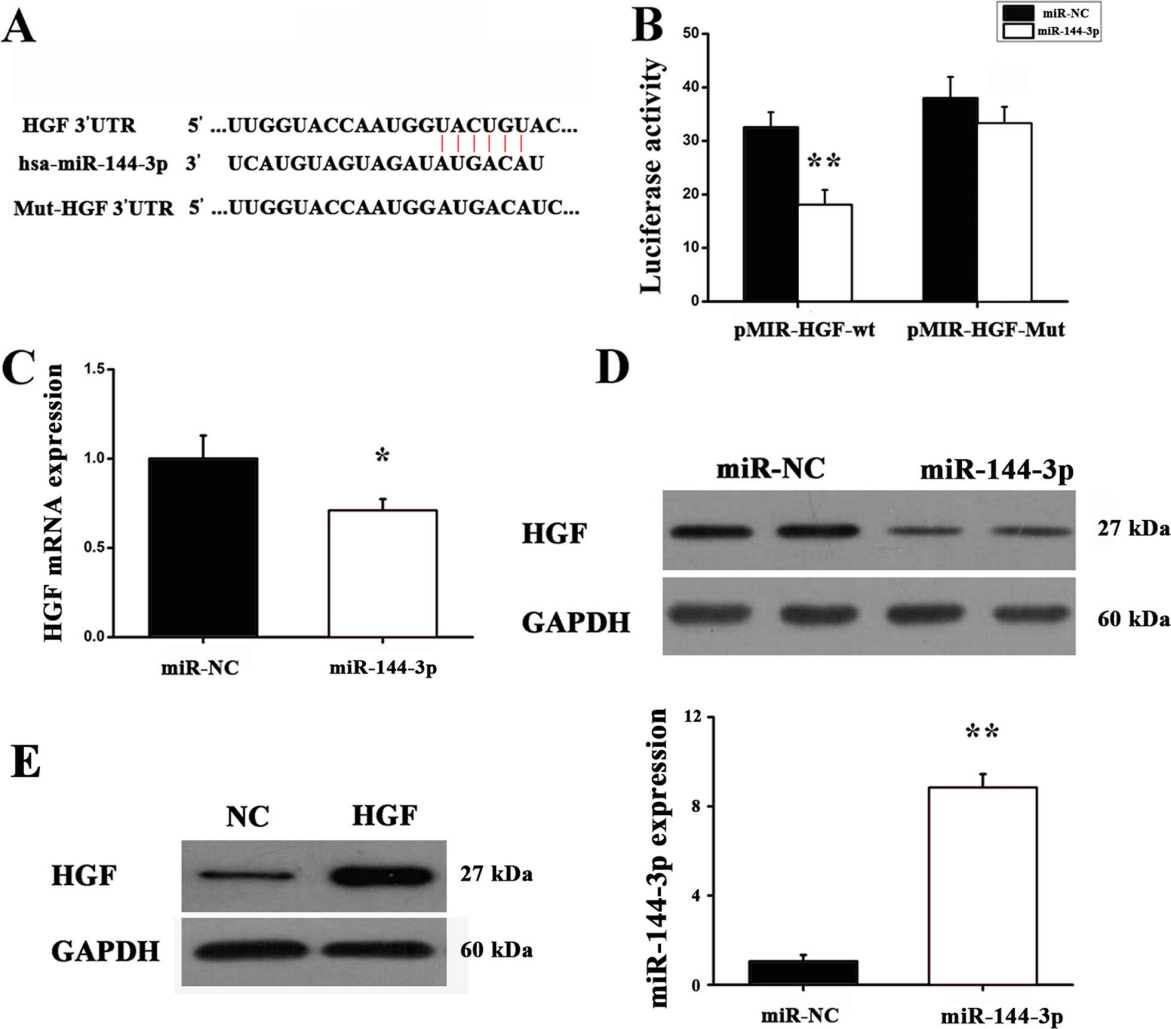
HGF is a direct target of miR-144-3p. **a** HGF 3′-UTR was a direct target of miR-144-3p. **b** Luciferase reporter assay detected the luciferase activity of cells co-transfected with the wild-type (WT) or mutant (Mut) HGF 3′-UTR reporter genes or negative control miRNA mimics (miR-NC). **P < 0.01 versus miR-NC. **c** RT-qPCR was used to quantify HGF expression at mRNA levels. **P < 0.01 versus negative control. **d** Western blot was used to measure HGF protein expression in A549 cells co-transfected with miR-144-3p or controls. **e** Western blot was used to detect the expression of HGF protein, and RT-qPCR was used to detect expression of miR-144-3p. **P < 0.01 versus miR-NC

Using bioinformatics analysis, hepatocyte growth factor (HGF) was recognized and identified as a putative target of miR-144-3p (Fig. 2a). In order to further assess whether miR-144-3p may bind to 3′-UTR of HGF or not, a luciferase reporter assay was performed. As indicated in the results, miR-144-3p strongly alleviated the luciferase activity of a reporter gene with the WT 3′-UTR. However, miR-144-3p had no impacts on luciferase activity of the mutant reporter gene, suggesting that miR-144-3p could directly bind to HGF 3′-UTR (Fig. 2b). To better understand whether miR-144-3p regulated HGF, RT-qPCR and western blot assays were performed. As demonstrated in the results, transfection with miR-144-3p resulted in markedly declining expression at not only the mRNA level, but also protein level (Fig. 2c, d). The transfection results are shown in Fig. 2e. Thus, it was implied that miR-144-3p could suppress HGF expression via direct combining with HGF 3′-Untranslated region.

### HGF expression in clinical specimens and various cell lines

**Fig. 3:**
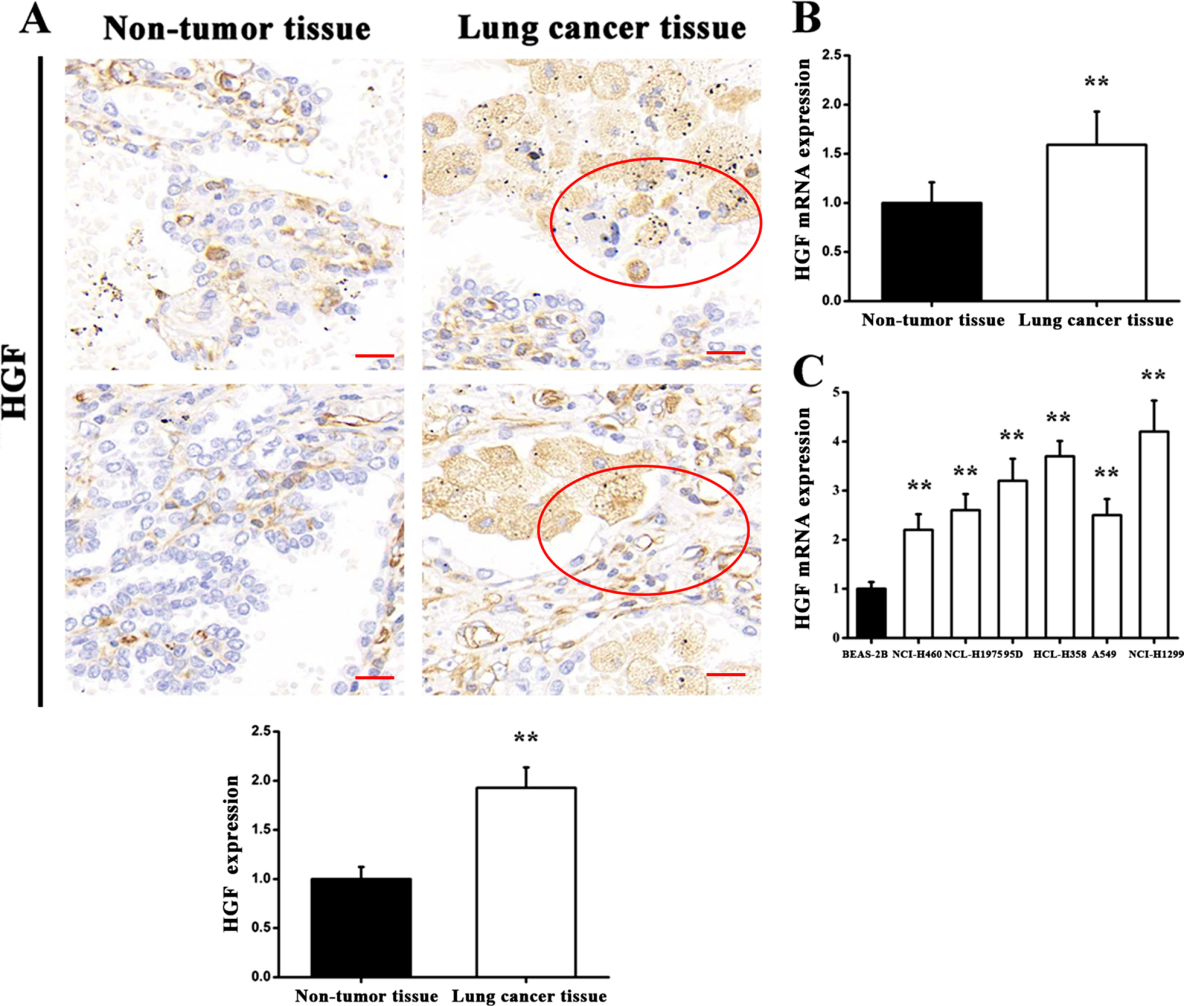
HGF expression levels in lung cancer tissues and various lung cancer lines. **a** Immunohistochemical staining was used to measure HGF protein expression. **b** RT-qPCR was used to quantify HGF expression at the mRNA level in tumor tissues and paired normal tissues. **c** RT-qPCR was used to analyze HGF expression in lung cancer cell lines and a normal lung cell line. **P < 0.01 versus controls. Scale bar = 50 μm

It has been demonstrated that HGF could significantly promote Small Cell Lung Cancer (SCLC) cell motility via the induction of tyrosine phosphorylation of numerous cellular proteins [22]. To try to further understand the role played by HGF in lung cancer, a variety of functional assays were conducted to detect HGF expression. Immunohistochemical staining demonstrated that HGF protein in lung carcinoma tissues exhibited elevated expression as comparing with adjacent normal tissues (Fig. 3a). Consistent with these results, an RT-qPCR assay revealed that mRNA levels of HGF in lung cancer tissues were significantly increased in comparison with non-tumor tissues from the same organ (Fig. 3b). Moreover, after detection of HGF expression in various lung cancer cell lines, it was shown that mRNA levels of HGF were higher in six lung cancer cell lines compared to normal lung cells. These results indicated that HGF may play an essential role in lung cancer initiation and development.

### Function of miR-144-3p in the phenotype of A549 andNCI-H1299 cells

**Fig. 4:**
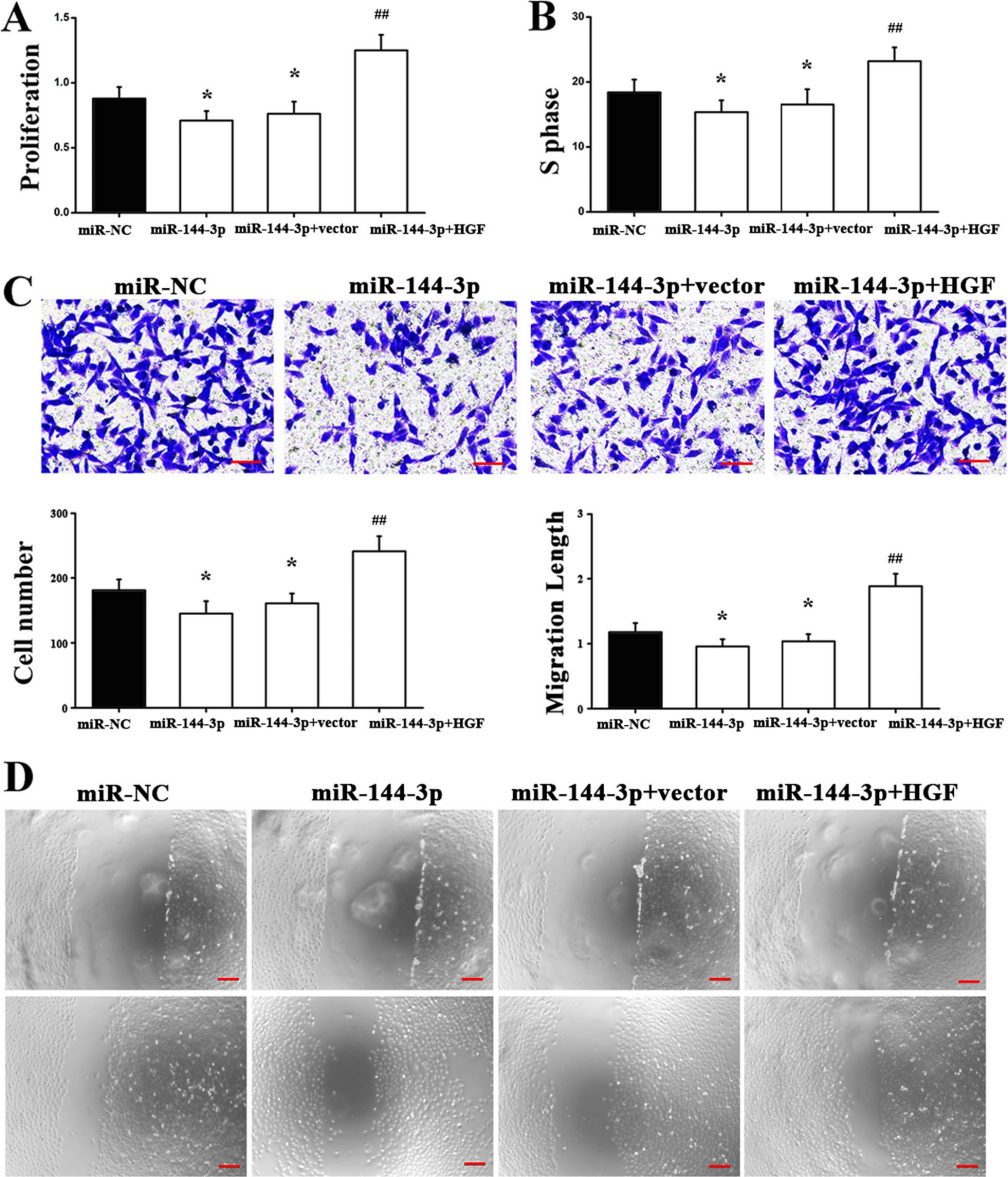
Function of miR-144-3p in A549 cells. **a** Proliferation of A549 cells transfected with control or miR-144-3p mimics together with HGF was detected by CCK-8 assay. *P < 0.05 versus negative control miRNA mimics (miR-NC). ^##^*P* < 0.01 versus miR-144-3p + Vector. **b** Flow cytometry was used to analyze the cell cycle. *P < 0.05 versus miR-NC. ^##^*P* < 0.01 versus miR-144-3p + Vector. Representative images and counts of cell migration following transfection with control or miR-144-3p together with HGF, **c** Transwell assay and **d** Wound-healing assay. *P < 0.05 versus miR-NC. ^##^*P* < 0.01 versus miR-144-3p + Vector. Scale bar = 25 μm. miR-NC, miR-144, miR-144 + vector and miR-144 + HGF need to be explained in a figure

**Fig. 5:**
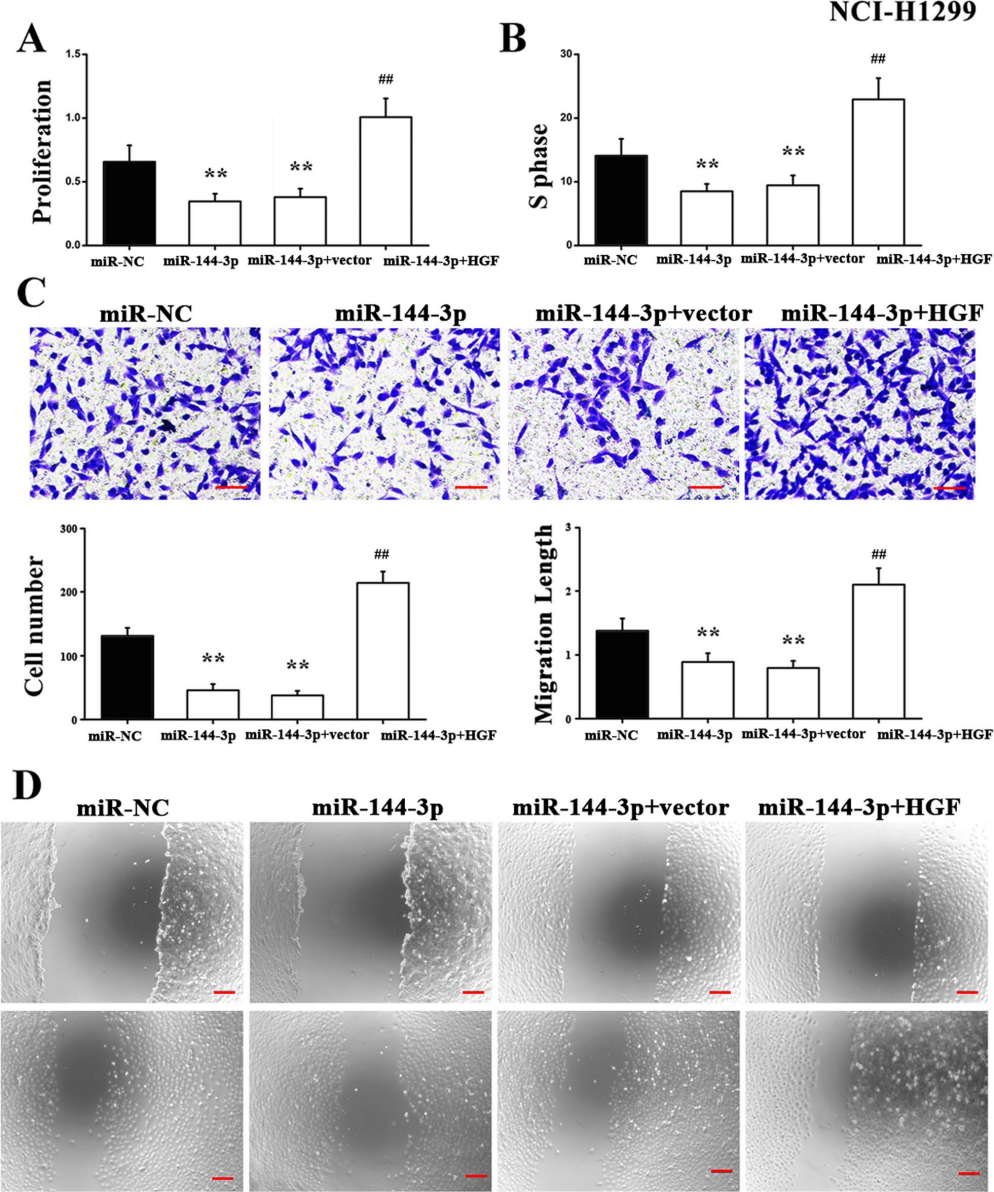
Function of miR-144-3p in the phenotype of NCI-H1299 cells. **a** Proliferation of NCI-H1299 cells transfected with control or miR-144-3p mimics together with HGF, as determined by CCK-8 assay. **P < 0.01 versus miR-NC. ^##^*P* < 0.01 versus miR-144-3p + Vector. **b** Flow cytometry was used to analyze the cell cycle. **P < 0.01 versus miR-NC. ^##^*P* < 0.01 versus miR-144-3p + Vector. Represented images and counts of cell migration following transfection with control or miR-144-3p mimics together with HGF, as detected by **c** Transwell assay and **d** Wound-healing assay. **P < 0.01 versus miR-NC. ^##^*P* < 0.01 versus miR-144-3p + Vector. Scale bar = 25 μm. miR-NC, miR-144, miR-144 + vector and miR-144 + HGF need to be explained in a figure

Based on the aforementioned results, an additional aim was to gain an insight about the underlying molecular mechanism by which miR-144-3p regulates lung cancer growth in vitro. It was indicated that HGF may serve as a target of miR-144-3p during development of lung cancer. Using a CCK-8 assay, it was found that miR-144-3p overexpression markedly inhibited A549 cell proliferation, whereas this effect could be reversed by treatment with HGF (Fig. 4a). In addition, flow cytometry analysis indicated that miR-144-3p made the proportion of cells in S-phase statistically significantly decreased. By contrast, such response was abrogated by overexpression of HGF (Fig. 4b). Furthermore, Transwell assay and wound-healing assay were conducted to evaluate cell migration, with results indicating that miR-144-3p exhibited an inhibitory impact on A549 cell migration, while transfection with HGF abrogated this response (Fig. 4c, d). The aforementioned results were repeated in NCI-H1299 cells, and we found that the results were consistent with A549 cells; miR-144-3p over-expression markedly suppressed proliferative ability of NCI-H1299 cells, while flow cytometry analysis indicated that miR-144-3p significantly reduced the proportion of cells in the S-phase. By contrast, these inhibitory effects were abrogated by HGF (Fig. 5a–d). In summary, these findings demonstrated that HGF, as a downstream gene, may be implicated in miR-144-3p regulation of the development of lung cancer. This suggests a novel pathway that could inform lung cancer diagnosis as well as its therapy.

### MiR-144-3p inhibits tumor growth in vivo

**Fig. 6:**
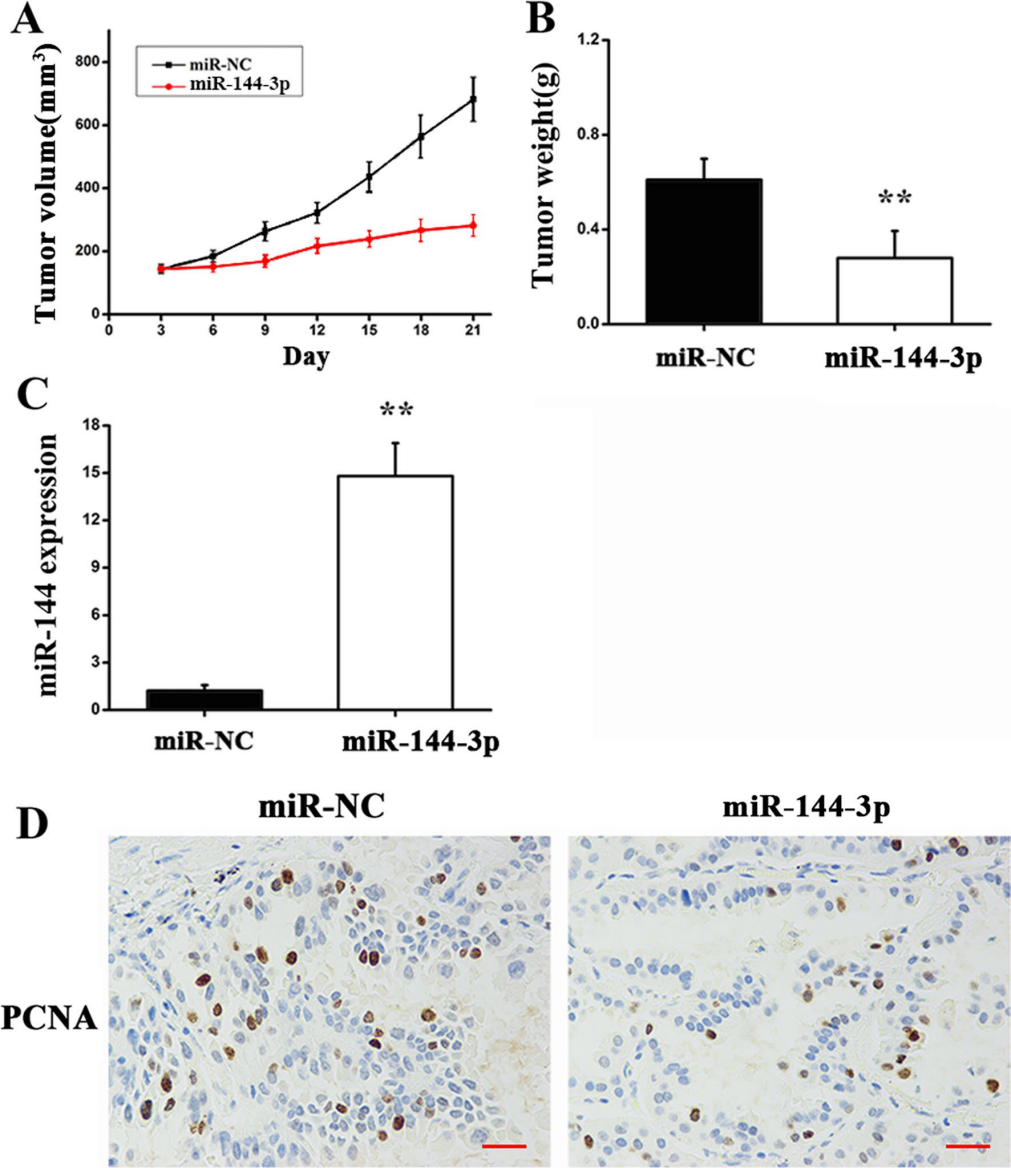
MiR-144-3p suppresses tumor growth in vivo. Nude mice accepted subcutaneous injection of A549 cells transfected with miR-144-3p mimics or control mimics on left flank of the mice. **a** Volume of Tumor. **b** Weight of Tumor. **c** RT-qPCR was used to quantify the miR-144-3p expression at the mRNA level in tumor tissues. **P < 0.01 versus controls. **d** Represented images of PCNA staining. Scale bar = 100 μm. miR-NC and miR-144 need to be explained in a figure

Constructing a mouse xenograft tumor model to further explore miR-144-3p’s function in vivo, the tumor volume was closely monitored after injection of A549 cells that were transfected with miR-144-3p mimics or control mimics. Treatment with miR-144-3p led to tumor weight and volume being statistically significantly reduced (Fig. 6a, b). In addition, the results of an RT-qPCR assay indicated that miR-144-3p expression at mRNA levels was higher in mice tumor tissues comparing with the controls (Fig. 6c). IHC analysis indicated that the expression of PNCA (a marker of cell proliferation) that was essential for DNA replication was suppressed by treatment with miR-144-3p (Fig. 6d). The above presented results indicated miR-144-3p exerted inhibitory effects on lung cancer.

## Discussion

MiRNAs are critical regulators involved in tumorigenesis [23, 24]. Epithelial-to-mesenchymal transition (EMT) appears to be crucial process in lung cancer cell invasion and metastasis, and Hepatocyte growth factor (HGF) is reported to make contribution to EMT in cancer development [25], which includes liver cancer [26], colorectal carcinoma [27], and prostate cancer [28]. As presented in this study, we found evidence that miR-144-3p exerts an inhibitory effect on lung cancer cell metastasis. More importantly, miR-144-3p/HGF signaling pathway could be an important regulator in EMT in lung cancer cell metastasis.

As observed in previous studies, aberrant downregulation of miR-144-3p has appeared in some types of cancers. For instance, miR-144-3p exhibited a significant down-regulation in osteosarcoma tissues and cell lines [29]. As for rectal cancer, miR-144-3p overexpression repressed rectal cancer cell viability, migration as well as proliferation [30]. Based on these results, it is indicated that miR-144-3p could affect tumor cellular proliferation and invasion in a variety of pathways and channels [31]. In keeping with this, we collected data from clinical lung cancer samples, and found that miR-144-3p expression was significantly reduced in lung tumor tissues compared to adjacent normal human lung tissues. Moreover, miR-144-3p expression was negatively associated with tumor tissues and tumor size. Similarly, in vitro observations indicated that miR-144-3p displayed a reduced expression in various lung cancer cell lines when comparing to normal lung cells, suggesting that miR-144-3p could play a critical role in lung cancer cellular proliferation.

Although the fact of miR-144-3p down-regulation in cancers has been demonstrated previously, the underlying mechanism of the involvement of miR-144-3p remains poorly understood. Using bioinformatics analysis, we found that HGF served as a potential target of miR-144-3p. As showed in luciferase reporter assay, miR-144-3p could directly bind to the HGF 3′-UTR. Moreover, RT-qPCR and western blot assays implied that miR-144-3p regulated HGF expression not only at mRNA level but also at protein level. HGF could activate the c-Met signal transduction cascade, drive both cancer cell invasion and metastasis and allow/proliferate/induce tumor cellular survival in bloodstream due to an absence of anchorage [8, 32–34]. As demonstrated in several studies, higher HGF levels in serum of patients suffering from mammary cancer are correlated with worse survival and distant metastasis [35–37]. It is worth noting that HGF levels appeared statistically significantly higher in patients suffering from SCLC compared to healthy controls [38]. HGF/c-met signal transduction appears to have a close relation with lung cancer occurrence, invasion and metastasis. Wang et al. [39] found c-Met exhibited a high expression in SCLC tissues and cells; c-Met knockdown could result in a reduction in both proliferation and invasiveness of lung cancer cells. In agreement with this, our results indicated that HGF exhibited high expression in lung cancer tissues and cell lines, suggesting HGF could be a regulator contributing to lung cancer development.

Growing evidence has indicated that HGF can induce c-Met activation, which promotes membrane ruffling, motility, migration and other tumor-related activities in lung cancer [40–43]. In addition, the molecular mechanism of HGF/c-Met is responsible for enhancing cancer cell invasion, proliferation, survival and morphogenesis via signaling pathways, such as PI3k/Akt, Ras/MAPK and JAK/STAT [44, 45]. In vitro, we observed the phenotype changes of A549 cells when transfected with HGF and miR-144-3p. As reported in a previous study, miR-144-3p may drive cell proliferation, migration as well as invasion in nasopharyngeal carcinoma by suppressing phosphatase and tensin homolog (PTEN) [46]. In response to knockdown of doublecortin and CaM kinase-like-1(DCAMKL-1), miR-144-3p level was increased, which in turn repressed pancreatic cancer EMT [47]. A549 cells are adenocarcinomic human alveolar basal epithelial cells, and have been regarded as testing grounds for brand new drugs—for instance, paclitaxel, docetaxel, and bevacizumab—both in vitro and in vivo. As found in this study, miR-144-3p attenuated proliferation and migration ability of A549 cells, as well as reduced the proportion of S-phase cells. This was also supported by an in vivo experiment, which totally complied the the regulations of the Ethics Committee of Ningde Normal University and the Guide for the Care and Use of Laboratory Animals. We found that miR-144-3p suppressed tumor growth and volume, and also inhibited proliferation in vivo. In contrast, the inhibitory function of miR-144-3p was reversed by overexpression of HGF. The shortcomings is that we have not inhibited the mRNA of HGF (by siRNA/shRNA) to identify the same phenotype as the overexpression of miR-144-3p, which might be detected in our future research. These observations support the proposal that miR-144-3p takes part in lung cancer development by targeting HGF. After conducting CCK-8, flow cytometry, Transwell and wound-healing assays in NCI-H1299 cells, we found that the experimental results were consistent with A549 cells.

## Conclusions

In summary, our work provides novel insight into real role and function of miR-144-3p expression in lung cancer cells. MiR-144-3p can function as a potential inhibitor to suppress lung cancer development by binding to HGF, and miR-144 could serve as an effective biomarker in lung cancer diagnosis.

## Data Availability

The data and materials in the current study are available from the corresponding author on reasonable request.
